# Peroxide of Hydrogen in Surgery

**Published:** 1882-11

**Authors:** 


### Peroxide of Hydrogen in Surgery.

From the *Lancet* we note that the powerful germicidal properties of peroxide of hydrogen, the *eau oxygenee* of the French, have led MM. Pean and Baldy to test its practical value as a surgical dressing for extensive wounds and ulcerations of various nature, as an injection into sinuses and cavities, such as the bladder, the nasal cavities, and also in the form of spray, as a substitute for carbolic acid spray, in major operations, such as ovariotomy. It was applied by means of compresses covered with an impermeable material to prevent evaporation. During the dressings a spray of it was employed. The preparation used was absolutely neutral in reaction, and contained four or six times its volume of oxygen; but for the injection of sinuses or closed cavities, a weaker solution was employed, containing only one or two times its volume of oxygen. The results obtained from more than a hundred cases have been most satisfactory, in grave as well as in trifling cases. Under the treatment, recent wounds made with the bistoury, or thermo-cautery, old wounds, even, when covered with gangrenous tissue, complicated with lymphangitis, or erysipelas, rapidly assumed a healthy aspect, granulating freely, with perfectly sweet, creamy pus. Chronic ulcerations rapidly cicatrized, and amputation wounds presented a strong tendency to heal by first intention. The general condition of the patients presented at the same time a marked improvement. The results are stated to have been quite as satisfactory as those obtained with carbolic acid. It has the additional advantage of being free from any toxic property, from any unpleasant odor, and of causing no pain. The results were especially satisfactory in some cases of varicose ulceration of the legs, intra-articular abscesses, ozæna, and purulent cystitis. In some remarks on the occasion of M. Pean's communication to the Académie des Sciences, M. Paul Bert, to whose investigations the French are indebted for most of their knowledge of the subject, pointed out that in the surgical use of this substance, its influence was exerted in two ways, first, by killing the organisms, and secondly, by continually liberating oxygen on the surface of the wound. He insisted on the care which must be taken to secure its purity, since most commercial specimens contain a considerable quantity of sulphuric acid.

